# Physical Exercise Program on Fall Prevention Using Technological Interface: Pretest Study

**DOI:** 10.2196/26196

**Published:** 2022-06-29

**Authors:** Mª Nilza Nogueira, Joana Silva, Isabel Nogueira, Maria Neto Pacheco, Joana Lopes, Fátima Araújo

**Affiliations:** 1 Escola Superior de Enfermagem do Porto Centro de Investigação em Tecnologias e Serviços de Saúde Porto Portugal; 2 Center for Assistive Information and Communication Solutions Fraunhofer Portugal Research Porto Portugal

**Keywords:** functional tests, fall assessment, exercices, older adults: games, technology

## Abstract

**Background:**

Prevention of falls among older adults has boosted the development of technological solutions, requiring testing in clinical contexts and robust studies that need prior validation of procedures and data collection tools.

**Objective:**

The objectives of our study were to test the data collection procedure, train the team, and test the usability of the FallSensing Games app by older adults in a community setting.

**Methods:**

This study was conducted as a pretest of a future pilot study. Older adults were recruited in a day care center, and several tests were applied. Physical exercise sessions were held using the interactive FallSensing Games app. Nurse training strategies was completed.

**Results:**

A total of 11 older adults participated. The mean age was 75.08 (SD 3.80) years, mostly female (10/11, 91%) and with low (3-6 years) schooling (10/11, 91%). Clinically, the results show a group of older adults with comorbidities. Cognitive evaluation of the participants through the Mini Mental State Examination showed results with an average score of 25.64 (SD 3.5). Functional capacity assessed using the Lawton Instrumental Activities of Daily Living Scale (overall score from 0-23, with lower scores reflecting worse capacity to perform activities) showed impairment in different instrumental activities of daily living (average score 14.27). The data collection tool proved to enable easy interpretation; however, its structure needed small adjustments to facilitate the data collection process. Despite the length of the questionnaire, its implementation took an average of 21 minutes. For the assessment of the prevalence of fear of falling, the need to add a question was identified. The performance of functional tests under the guidance and presence of rehabilitation nurses ensured the safety of the participants. The interactive games were well accepted by the participants, and the physical exercises allowed data collection on the functionality of the older adults, such as the number of repetitions in the tests, range of movement (angle), duration of the movements, and execution of each cycle. Concerning the training of the nurses, it was crucial that they had experience with the platform, specifically the position of the chair facing the platform, the position of the feet, the posture of participants, and the use of sensors.

**Conclusions:**

In the future pilot study, the researchers point out the need to design a study with mixed methods (quantitative and qualitative), thus enriching the study results.

## Introduction

In community settings, fall episodes are highly prevalent among the population of older adults [1,2]. Regardless of the severity of the related injuries, the impact on health and quality of life of older adults and their families can be significant since they often trigger and accelerate a cycle of restrictions and barriers leading to the dependency of the older person for activities of daily living [3-5]. Evidence suggests that the frequency of falls increases with age and degree of fragility [6] and that the presence of risk factors directly influences the risk of fall [7,8].

A structured and standardized screening and assessment of the risk of fall in older adults contribute to its prevention and reduction and are central to the design of the intervention and risk monitoring [7].

Changes in gait and balance are factors that have been strongly associated with the outcome of fall in older adult population, and rapid tests, such as the 30-Second Chair Stand Test (30CST) [9], the 4-Stage Balance Test (4SBT) [10,11] and the Timed Up and Go (TUG) test [7,12], are recommended for their assessment.

Evidence-based fall prevention programs have demonstrated a significant reduction in fall risk, falls, and related injuries in older people in a community setting [13,14]. An exercise program with proven effectiveness in preventing falls is the Otago Exercise Program (OEP), designed at the University of Otago Medical School [15-21]. The focus of the OEP is to improve strength and balance with a simple, affordable, and easy home-implemented solution for 12 months, monitored by a health professional through monthly telephone interviews and biannual home visits.

Recent evidence has strived to integrate technologies into physical exercise programs that have shown a positive effect in adherence and overcoming barriers to exercise, as well as improvements in physical functioning [22,23]. Some technological solutions to facilitate the process of monitoring and fall prevention have already been developed in Portugal, such as the FallSensing Screening and FallSensing Games apps, designed by Fraunhofer Center for Assistive Information and Communication Solutions (AICOS) Portugal.

The FallSensing Screening app uses inertial measurement units (IMUs) to extract information about the user’s movements, using these data to characterize the movement; it then uses metrics calculated after processing the sensor signal, obtained during the execution of the functional tests performed by the user. The IMU, composed by a triaxial accelerometer, triaxial gyroscope, and triaxial magnetometer, was used to acquire inertial data during the exercises at 50 Hz. Data were transmitted using Bluetooth Low Energy wireless technology to a main computer where the processing occurs. The interactive FallSensing Games app, based on the OEP, aims to improve physical functionality and is also used as a motivator for participants who perform the exercises.

Therefore, there is an excellent opportunity and a need to develop new user-tailored solutions supported on more robust and valid fall risk predictive models and good clinical practice in fall prevention [6]. Technological solutions need validation in a clinical context, through methodologically robust experimental studies.

In research, pretesting is an essential stage before the pilot study because it allows for identifying weaknesses in the development of measurement instruments (structure, content, semantics) to determine the potential respondents’ difficulty in interpreting the questions and complexity of the evaluation process. In addition, it enables benchmarking and training procedures and standardizes the modus operandi in data collection, thus contributing to improving the reproducibility and accuracy of measurements [24,25]. This study aims to test the data collection procedure, train the team, and test the usability of the FallSensing Games app by older adults in a community setting.

## Methods

### Study Design

A pretest study was performed for the successful implementation of a future larger pilot study. Two research centers were involved in the project, the Nursing School of Porto (ESEP) and the Fraunhofer AICOS Portugal.

### Participants

Participants were recruited in one of the day centers in western Porto city. For the realization of the FallSensing Games, a minimum of 6 participants was required, but in this study the sample included 11 older adults.

The inclusion criteria were being aged 65 years or older, living at home, walking independently, not presenting with cognitive impairment according to the Portuguese version of the Mini Mental State Examination (MMSE) [26], not having severe visual or hearing impairment, signing informed consent, and presenting moderate to high risk of falling (assessed through 4 short questions with a dichotomous answer option).

Participant exclusion criteria included having chronic or acute illness conditions for which exercise is contraindicated; ever having hip or knee surgery or having a history of lower limb fractures in the last 12 months; having participated in physical exercise programs in the last 12 months; having participated in another research study; or having a final MMSE score below 22 (with up to 2 years of school), below 24 (3 to 6 years of school), or below 27 (7 years or more of school).

### Materials

**Figure 1:**
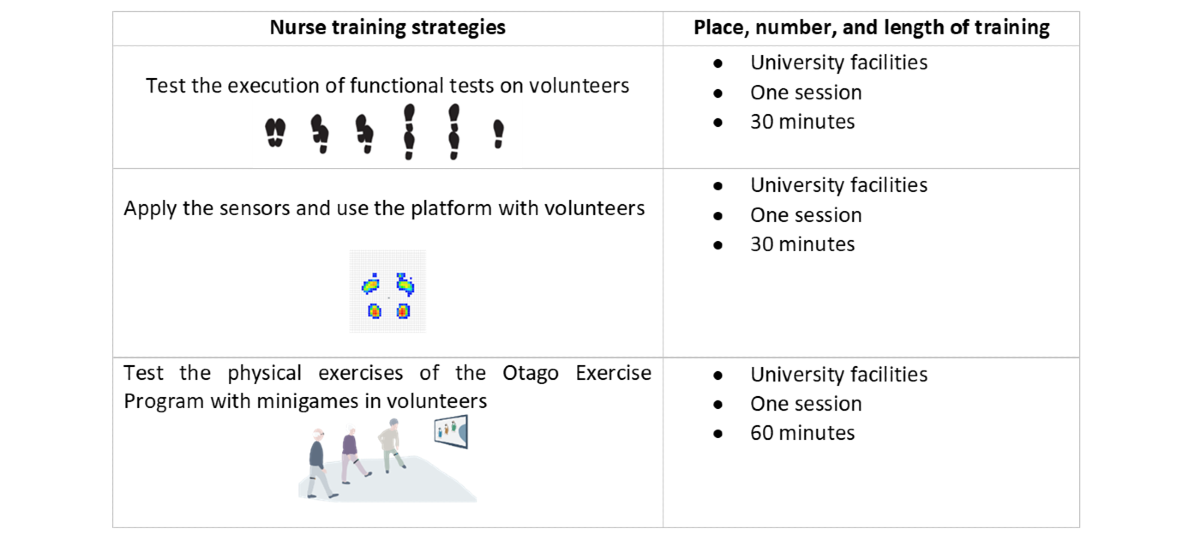
Training procedures.

After selection criteria application, data were collected by the main researchers, and functional testing was performed by two rehabilitation nurses who had received training sessions. In accordance with the best practices recommended for clinical research, the main researcher ensures that their team is trained to implement the different procedures at the different stages of the investigation process. Thus, the training of rehabilitation nurses was performed by the principal investigators. A meeting was held with all the investigators and rehabilitation nurses to present the investigation plan, provide the study dossier (research project, assessment instruments for functional testing instruments, and Otago exercise manual), introduce technologies, explain data collection procedures, and review communication techniques. In the end, there was time for clarification. Subsequently, a training session was held (Figure 1).

**Figure 2:**
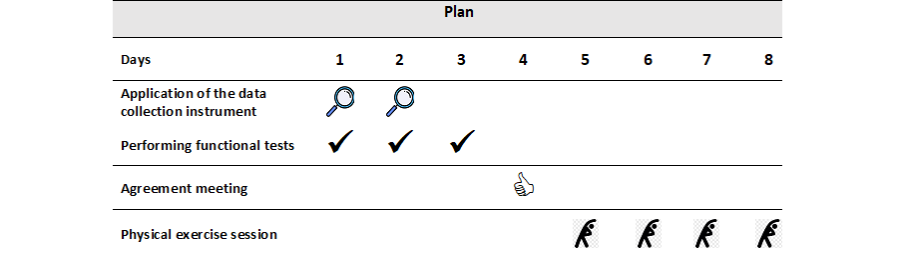
Study plan.

The training procedures took place for a week, covering use of the data collection instrument and performance of functional tests and physical exercises sessions. Among investigators, after consensus meetings, 2 researchers used the data collection instrument, independently and randomly among the participants, in similar spaces. The monitoring of the instrument application time and use of field notes to document difficulties and other observations, such as opinions made by the respondents, were the resources used to assess the applicability of the data collection instrument. Before moving on to the physical exercise sessions, the researchers met to analyze and decide by consensus the questionnaire items identified as needing improvement. Participants performed the functional tests on 3 consecutive days to avoid interfering with the activities previously planned by the day center (Figure 2).

### Instrument

The data collection instrument included sociodemographic, clinical, and functional evaluation; fear of falling, and the acceptance of technology. Lower limb strength and muscle resistance were the functional variables assessed through the 30CST; mobility was evaluated by the TUG test (normal step). These functional tests and the 4SBT allowed evaluation of the risk of fall, which was also assessed with the Fall Risk Screening Tool. The functional capacity for activities of daily living was assessed using the Lawton Instrumental Activities of Daily Living Scale (IADL), fear of falling using the Falls Efficacy Scale–International (FES-I), and acceptance of technology by participants and health professionals by the System Usability Scale (SUS). The domains of the instrument are presented below.

#### 30CST Instrument

The performance in the 30CST is used as a measure of the strength and muscle resistance of the lower limbs, specifically the extensor muscles of the knee [27,28]. It is a quick test without a dynamometer, training, or special equipment, which allows evaluating the strength of the lower limbs by counting the number of times the individual stands and sits in 30 seconds [9-30]. More strength in the lower limbs is associated with better balance [9-30], and the functional improvement of older adults after a fall prevention program will be manifested by a greater number of repetitions in 30 seconds in the posttest assessment [14]. The results have shown good psychometric qualities [9-30].

#### TUG Test (Normal Step)

Since mobility assessment of older adults is a central component in the geriatric assessment [31], the TUG test is proposed to evaluate the clinical utility of the timed stand and walk test. This test measures in seconds the time an individual takes to stand from a chair, walk a distance of 3 meters, return, and sit back in the chair. These authors reported that time spent on the TUG test performance was related to scores on the Berg Balance Scale (*r*=–0.72) and the walking speed (*r*=–0.55) and Barthel Activities of Daily Living Index (*r*=–0.51) scores. Individuals who completed the test in less than 20 seconds were independent in transferring, and individuals who completed the test in more than 30 seconds tended to be dependent on this task.

The TUG test has been widely referred to and used [12] as a screening test to assess the risk of fall in older adults in community settings, namely through the guideline of the American Geriatric Society and British Geriatric Society and in the US Centers for Disease Control and Prevention (Stopping Elderly Accidents, Deaths, and Injuries [STEADI initiative]).

A prospective design conducted to evaluate the predictive ability of the TUG test for future falls and estimate the best cutoff point of the test pointed to 12.6 seconds, with the corresponding values of sensitivity (30.5%), specificity (89.5%), positive predictive value (46.2%), and negative predictive value (81.4%) [12]. The researchers who conducted the study emphasized the high specificity (89.5%) and high negative predictive value (81.4%) to a cutoff point of 12.6 seconds as a support for the clinical utility of this test in older adults at high risk of falling. Researchers reported that after a fall prevention program, performing the test in less time is indicative of improvement [14]. In Portugal, this test has been used in several studies [32,33].

#### 4SBT Instrument

The balance tests were conceptually developed to track balance impairments [11,34,35] placing the older adults at risk of falling [10,11]. More specifically, the 4SBT is used to track impairment in the static balance of older adults. Several authors have found the test with an excellent performance in test-retest reliability (*r*=.97) and interrater reliability (Κ=.92) [10,36]. The success of fall prevention programs is measured by comparing the positions achieved in 10 seconds in the pre- and postprogram evaluation [14]. The final score will be the number of positions successfully completed for 10 seconds without losing balance. The older adults who cannot maintain position 3 for 10 seconds have a high risk of falling [37].

#### IADL Instrument

The IADL assesses the level of independence of older adults in performing activities of daily living, which integrate day-to-day tasks such as using the telephone, shopping, preparing food, housekeeping, washing clothes, using transport, preparing medication, and handle finances. It is an easy-to-administer tool that can be used with older adults in a community and hospital setting [38-40].

In this study, the Portuguese version [41], which uses the same items as the original version but applies a polychotomous score (0, 1, 2, 3, or 4) instead of the original dichotomous score (0 and 1), was used, allowing for a better description of a person’s ability to perform the tasks, giving each response option a different score. The total score of the scale varies from 0 to 23, with a lower score corresponding to worse performance. In the validation study, the instrument showed good metric qualities to be applied in a community setting.

#### FES-I Instrument

The fear of falling among older adults is an expressive problem and highly relevant because it is associated with adverse effects on mobility and quality of life [42-44]. One of the instruments used to evaluate this construct is the FES-I [45]. Its adaptation to different languages and cultural contexts (following the protocol recommended by the Prevention of Falls Network Europe Group), has allowed the instrument to be widely applied and the results compared in different populations and contexts. The FES-I version is an instrument that incorporates some daily activities that are a little more complex than those of the original version and others more focused on the social life of older adults as a way to overcome some weaknesses identified in the original version. For each of the 16 items, the answer option is based on a 4-point Likert scale (1=not at all worried; 2=somewhat worried; 3=moderately worried; 4=very worried). The instrument has shown validity, reliability, and comparability across cultures, so it is recommended for research practice and the clinical context, namely in fall prevention programs for older adults population [46].

In this research, the FES-I version validated for the Portuguese population [47] showed excellent internal consistency (α=.98) and test-retest reliability (intraclass correlation coefficient 2.1=0.999). Concurrent validity, assessed using the Activity-Specific Balance Confidence Scale, presented results indicative of good concurrent validity (*r_s_*=–0.85; *P*<.001). Considering the global results, the authors consider the Portuguese version of the FES-I a reliable and valid measure to assess the fear of falling among the Portuguese older adult population living in the community.

#### SUS Instrument

To validate the acceptance of the technology by participants and health professionals, the responses of the SUS was analyzed. This rapid test evaluates the usability of a certain product or service [48]. This test has several features that provide a good assessment of the overall usability, such as the flexibility to evaluate interface technologies, interactive voice response systems [49], hardware platforms used in more traditional computer interfaces, and websites. Ease and speed of use (by both participants and system administrators), ease of operation of scoring, and the free access characteristic are also advantages.

The original SUS instrument consists of 10 statements that are scored on a 5-point Likert scale (1=strongly disagree to 5=strongly agree) [48]. The final score can vary from 0 to 100 points, with a better score indicating better usability [49], and the final score needs to be considered following the instructions of the original instrument because statements switch between positive and negative. A study conducted at the national level performed a psychometric analysis of the tool intending to translate, culturally adapt, and contribute to the validation of the European Portuguese version of the SUS [50].

### Procedures

#### Technology Platform

The technology platform uses mobile app inertial sensors to extract information on movement performed by the participant and related characterization. This platform measures pressure distribution at 50 Hz and comprises 1600 pressure sensors (10 mm × 10 mm) with maximum value of 100 N/sensor. The size of the active area of the pressure platform is a square matrix of 40 cm × 40 cm. Voltage data are converted with an 8-bit A/D converter and transmitted via USB to the main computer. The risk of fall is then determined from parameters calculated after processing the signal from the inertial sensors during the execution of functional tests such as walking, sitting, and standing.

#### Games

The interactive FallSensing Games app is based on the OEP and the use of inertial sensors to monitor the movements performed by the participants during these exercises. To interact with the characters and achieve the objectives of each game, participants must perform the suggested movements correctly. Monitoring the movements of participants in each game allows us to assess the evolution of physical capacity and extract parameters related to functional capacity.

The FallSensing Games app comprises 3 minigames, with each minigame comprising 2 to 3 exercises from the OEP. The composition of the minigames is as follows [29]:

- Minigame 1 includes knee bends and sit-to-stand exercises monitored with a sensor on the thigh.
- Minigame 2 includes lateral hip abduction (side hip), frontal knee extension (front knee), and backwards knee flexion (back knee) monitored with the sensor on the ankle.
- Minigame 3 includes calf and toe raises monitored with sensors on the top of the foot.

#### Physical Exercise Session

The Otago physical exercise session, supported by interactive games, was implemented by the rehabilitation nurses in the day centers. Participants were divided into 2 groups. In the physical exercise session, after the demonstration, the rehabilitation nurse started with the warm-up exercises suggested by the OEP followed by exercises to strengthen the lower limbs and improve balance and stability and finally the relaxation phase, with stretching exercises. For the implementation of the physical exercise session, we used the following material resources: (1) room with free space (at health centers), (2) computer and television/projector, (3) wearables (IMUs) with loaders and fixing tapes, and (4) a pressure platform.

### Ethics Approval

The pretest study was approved by the Health Ethical Committee from ESEP (annex 5 to document no. 4/2019). All participants were informed and provided informed consent in duplicate (one copy for participant and one copy to investigator) before enrolling. Participants were informed about the confidential information protection, the right to study withdrawal, data anonymity, and the likelihood of study publication.

### Data Analysis

SPSS (version 26.0, IBM Corp) software was used for statistical analysis. The univariate descriptive analysis was applied to describe data supported on measures of central tendency and dispersion.

## Results

### Descriptive Information

The pretest was conducted on 11 participants with a mean age of 75.08 (SD 3.80) years, mostly female (10/11, 91%) and with low (3-6 years) schooling (10/11, 91%). Clinically, the results show a group of older adults with comorbidities who portray the epidemiological profile of chronic disease, with high expressiveness of hypertension, osteoarticular disease, and urinary incontinence. In this sample, despite the high prevalence of osteoarticular disease, only 2 older adults used walking aids. More than half (6/11, 55%) of participants reported depression. This clinical pattern was accompanied by drug regimens that integrate mostly 4 or more drugs (10/11, 91%). Balance impairment or difficulty in walking was referred to by 64% (7/11) of participants. More than half (6/11, 55%) presented a high risk of falling, due to recurrent falls in the last 12 months.

Cognitive evaluation of the participants using the MMSE showed results with an average score of 25.64 (SD 3.5), consistent with a mild degree of impairment for participants with a low level of education. Functional capacity assessed using the IADL (overall score varying from 0 to 23, with lower scores reflecting worse capacity to perform activities) showed impairment in different activities of daily living (average score 14.27). The results of the descriptive analysis for sociodemographic and clinical variables are presented in Table 1.

Concerning the fear of falling, the results showed that the activities in which participants reported higher levels of fear (response options: 3=moderately concerned and 4=very concerned) were walking on slippery surfaces (7/11), going up and down stairs (6/11), and walking on uneven surfaces and walking up and down slopes (5/11 for both). In the self-care dressing/undressing, shopping, and walking in the neighborhood, 4 older adults were identified with the response option 3 or 4 on the Likert scale. The response option equal to 2 (a little worried) was expressed by 10 out of 11 participants for a variable number between 1 to 6 activities.

**Table 1:** Participant characteristics.

Characteristics			Value
**Age (years), mean (SD)**			75.09 (3.80)
	65-74, n (%)	3 (27)	
	75-84, n (%)	8 (73)	
Gender (female), n (%)			10 (91)
**Marital status, n (%)**			
	Married	2 (18)	
	Single	2 (18)	
	Divorced	2 (18)	
	Widowed	5 (45)	
**Education (years), n (%)**			
	0-2	1 (9)	
	3-6	10 (91)	
**Comorbid health conditions (yes), n (%)**			
	Arterial hypertension	8 (73)	
	Osteoarticular disease	8 (73)	
	Urinary incontinence	8 (73)	
	Depression	6 (55)	
	Vertigo syndrome	6 (55)	
	Diabetes mellitus	5 (45)	
	Vision changes	4 (36)	
Daily medication consumption (≥4), n (%)			10 (91)
MMSE^a^ score, mean (SD)			25.64 (3.50)
IADL^b^, mean (SD)			14.27 (7.14)
Walking difficulties/balance compromised (yes), n (%)			7 (64)
Walking aids (yes), n (%)			2 (18)
**Falls (yes), n (%)**			
	High risk	6 (55)	
	History of falls (last 12 months)	6 (55)	
	Recurrent falls	6 (55)	
	Indoor falls	4 (67)	
	Health care need after falls	2 (33)	

^a^MMSE: Mini Mental State Examination

^b^IADL: Lawton Instrumental Activities of Daily Living Scale

### Data Collection Procedure

In general, at the interview stage no difficulties of interpretation were identified that could make it difficult to answer the questions of the data collection instrument; however, for the FES-I assessment both researchers needed to frequently recall the Likert scale in use. In the consensus meetings, it was found in the field notes of the researchers that the behavior of the older adults in the assessment of fear of falling are indicative of increased difficulties in interpreting the request and choosing the answer option. This fact showed the need to evaluate this construct in a simpler way that also allowed distinguishing the participants regarding the level of fear of falling. After research, it was decided to include a single-item question “Are you afraid of falling?” with the same ordinal answer option (not at all worried, a little worried, moderately worried, very worried) in the definitive questionnaire for the future pilot study. According to some authors, there is not enough evidence that more complex measures consisting of several items better assess the fear of falling into this population range than single item questions [51-53].

In the 30CST test, an average of 4.9 repetitions was obtained. The average time of the TUG test was 21.9 seconds, with 12.6 seconds being used as a cutoff point, less time in the test performance means better functional condition. Finally, in the performance of the 4SBT, all participants were able to perform positions 1 and 2 with their eyes open, but only 55% (xx/xx) were able to achieve position 3 (Table 2).

**Figure 3:**
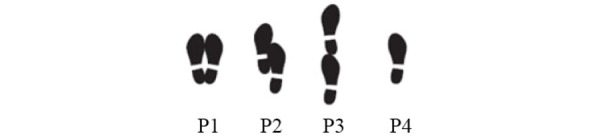
Number of foot positions in 4-Stage Balance Test, with eyes open.

During the functional tests, the participants presented difficulties in the execution of the instruction given for the 4SBT test, despite the previous demonstration of the 4 foot positions performed by both rehabilitation nurses under the supervision of the investigators (Figure 3).

During the 4SBT functional test, the older adults showed a behavior of constantly searching for support in the surrounding environment (people, walls, chairs). This fact is reported in the literature on falls in elderly populations as indicative of fear of falling or low perception of self-efficacy to perform the task.

The average time to complete the data collection tool—sociodemographic data, clinical and drug consumption history, IADL, and FES-I—was 21 (SD 2.62) minutes. Before starting data collection, each of the researchers reminded the participants of the study objectives and the possibility of being able at any time to express their willingness to withdraw without any negative implication.

**Table 2:** Functional test results.

Tests		Value	Minimum-maximum
30CST^a^, mean (SD)		4.9 (3.315)	0-11
**TUG^b^**			
	Duration (seconds), mean (SD)	21.90 (5.74)	13.96-31.38
	≥12.6 seconds, n (%)	100	—^c^
**4SBT^d^ (4 foot positions), n (%)**			
	Position 1	100	—
	Position 2	100	—
	Position 3	55	—
	Position 4	9	—

^a^30CST: 30-Second Chair Stand Test.

^b^TUG: Timed Up and Go.

^c^Not applicable.

^d^4SBT: 4-Stage Balance Test.

### Train the Team

The training of nurses was meant to standardize the application of functional tests, guidance in games and physical exercises, and interactions with the older adults through appropriate communication techniques and security measures. Regarding the use of technology, the training of nurses allowed validation of the correct use and placement of sensors as well as the correct use of the platform.

#### Functional Tests Application Procedure

Before the functional tests application procedure, there was a need to establish a relationship of trust between the nurses and participants. The tests were explained and then demonstrated. Special attention was given to the nurses’ position toward older adults, tone of voice, rhythm of explanation, and nonverbal communication.

**Figure 4:**
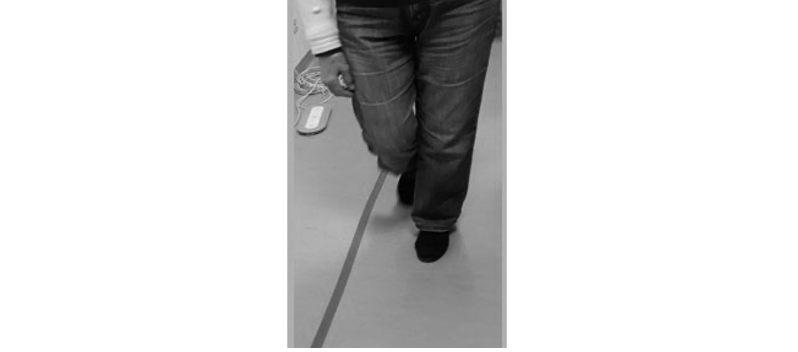
Timed Up and Go test execution path, marked with colored ribbons.

In particular, for the 4SBT it was important to measure the position of the chair facing the platform, the position of the feet, and the posture of older adults on the platform. For both tests (4SBT and TUG), the position of the nurse beside older adults during the test execution proved to ensure the safety of the participants. To mark the path of the execution of the TUG test, colored ribbons were placed on the floor, which were identified by the participants as providing good assistance (Figure 4).

**Figure 5:**
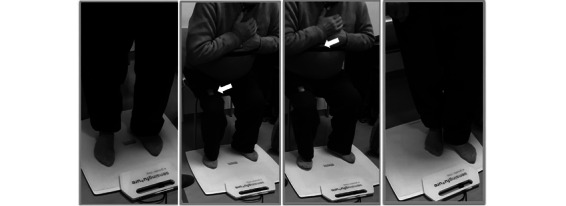
Anatomical position of sensors and foot positions inside the platform.

Regarding the use of technological devices, it was necessary to check the position of the sensors, both in the anatomical location and in their local adjustment (avoiding discomfort for older adults or coming loose in order to obtain correct readings and avoid repeating the test several times (Figure 5).

#### Physical Exercise Session

The physical exercise session using the FallSensing Games app led to adjustments in its implementation: (1) maintenance of a minimum distance between the participants’ chairs to avoid touching each other during the shoulder abduction exercise, (2) synchronization between the material resources (TV/computer) and the start of the warm-up exercises, (3) presence of the nurse near the participants to adjust the correct use of the elastic bands, and (4) adaptation of the nurses’ paralanguage (tone of voice) to the sound volume of the games.

#### Minigames

For each exercise composing 1 of the 3 minigames, the wearable inertial sensors allow us to extract 3 relevant metrics, such as the range of motion angle (angle), range of motion duration (cycle time), and number of repetitions (nr_cycles). Considering that each exercise requires a specific number of repetitions defined according to the OEP, each of these metrics will be computed for each repetition. For example, if we consider the knee bending exercise, each person should perform 10 repetitions of the exercise, allowing us to compute the angle and duration of each repetition and also count the number of performed repetitions. Given that each participant performed each 1 of the 3 minigames, we have computed the mean of each metric for each participant. Table 3 presents the values for each metric averaged for all the participants and its standard deviation.

For minigame 1, each participant performed on average 6 repetitions of sit-to-stand and 23 repetitions of the knee-bending exercise. For minigame 2, each participant performed on average 25 repetitions of knee extension, 25 repetitions of knee flexion, and only 13 repetitions of hip abduction (side hip) exercise. For minigame 3, each participant performed on average 15 repetitions of calf raises and 13 repetitions of toe raises.

In sum, each participant performed on average more repetitions of each exercise than suggested in the OEP due to the gamification of these exercises in the minigames, which requested the participants to perform more repetitions to accomplish a higher game score. This can be seen as a positive effect of the gamification of the Otago exercises. Another relevant outcome is the retrieval of range of motion–related metrics, as the angle and duration of movements, which can only be quantified when using wearable sensors as opposed to traditional observational programs.

**Table 3:** Minigame metrics.

Game number and inertial sensor metric		Value, mean (SD)
**Minigame 1**		
	Sit_to_stand_angle	64.43 (22.29)
	Sit_to_stand_cycle_time	3.20 (1.46)
	Sit_to_stand_nr_cycles	6.33 (4.54)
	Knee_bends_angle	35.67 (14.61)
	Knee_bends_cycle_time	2.19 (0.82)
	Knee_bends_nr_cycles	22.58 (14.44)
**Minigame 2**		
	Knee_extension_angle	80.02 (21.80)
	Knee_extension_cycle_time	2.02 (1.37)
	Knee_extension_nr_cycles	25.00 (10.39)
	Knee_flexion_angle	80.98 (17.84)
	Knee_flexion_cycle_time	1.90 (0.98)
	Knee_flexion_nr_cycles	24.56 (10.74)
	Side_hip_angle	50.03 (26.11)
	Side_hip_cycle_time	2.45 (1.11)
	Side_hip_nr_cycles	13.00 (5.02)
**Minigame 3**		
	Calf_raises_angle	35.72 (22.92)
	Calf_raises_cycle_time	3.46 (4.72)
	Calf_raises_nr_cycles	14.75 (9.85)
	Toe_raises_angle	29.48 (24.21)
	Toe_raises_cycle_time	2.64 (1.86)
	Toe_raises_nr_cycles	13.42 (5.98)

### Test the Usability of the FallSensing Games App

As for participant satisfaction using the SUS, the results showed that out of the participants who responded, 50% (5/10) assessed the usability of the technology as acceptable, 30% (3/10) expressed good satisfaction, and 20% (2/10) considered the usability of the technology as problematic. One participant did not respond.

In addition to this quantitative analysis, field notes were collected on satisfaction expressed by the participants, who voiced their satisfaction with the games, participation in team games, and repetition of the activity’s animated penguins. Statements from participants included “I had never made a game looking at penguins,” “I didn’t realize that time was passing,” “I even forgot the pains,” “I even laughed a little bit,” and “You need to come here more often.” During the stay at the day center, we observed the acceptance of the games, both for the ease of integration in the activities of older adults and for the ease with which older adult followed the games.

## Discussion

### Principal Findings

#### Test the Data Collection Procedure

From the nurses’ perspective, due to the speed and consistency of the participant answers, the data collection tool proved to enable an easy interpretation. However, its structure needed small adjustments to facilitate the data collection process. Despite the length of the questionnaire, its implementation took an average of 21 minutes. For the assessment of the fear of falling, the need to add a question was identified to clarify whether the participant was afraid of falling. The performance of functional tests by the participants under the guidance and presence of rehabilitation nurses ensured the safety of the participants.

#### Train the Team

Concerning the training of the nurses, it was crucial that they had experience with the platform, specifically the position of the chair facing the platform, the position of the feet, and the posture of the participants on the platform, which allowed adjustments to minimize errors in the functional test assessment. At the same time, the use of sensors and their anatomical position and adjustment allowed us to understand that the way to hold them needs to be improved.

#### Test the Usability of the FallSensing Games App

Regarding the games, we can point out 2 aspects. On the one hand, each participant performed on average more repetitions of each exercise than suggested in the OEP to achieve a higher game score. On the other hand, obtaining metrics related to range of motion, such as the angle and duration of movements, was only possible with the use of wearable sensors. The easy integration of games in the activities of the older adult care center and the ease of the older adults in following the games corroborates the results presented by previous research [29].

### Limitations

As limitations of the study, we highlight the (1) small sample size; (2) absence of an observation grid of the participants’ behavior during the performance of the functional tests and games, which could, in a future pilot study, reflect the realism of the situation under study; (3) spontaneous appreciation of the participants, expressed by the contentment and appreciation of the moments spent together, showing the researchers the need to collect this experience in a planned and rigorous way, namely the feelings and emotions of the participants; and (4) concern to prepare the team of nurses for the application of functional tests, use of the platform and sensors, and physical exercise session with the games led to some aspects being neglected, namely the possibility of incorporating qualitative component into the study.

Therefore, in the future pilot study, the researchers point out the need to design a study with mixed methods (quantitative and qualitative), thus enriching the study results. The researchers, intend to use qualitative methods, such as focus group, for the participants, which can enrich the exchange of experiences during the games and nonparticipant observation, with the use of an observation grid, which can favor the collection of information on the correct execution of the Otago exercises.

Regarding the sample size, the recruitment can be improved by incorporating more day centers and a longer period for data collection.

Despite the limitations of the pretest study and results, this study aims to contribute to the practice of professionals in clinical and research contexts, given the scarcity of information on this relevant stage in experimental/quasi-experimental studies.

## Abbreviations

30CST: 30-Second Chair Stand Test
4SBT: 4-Stage Balance Test
AICOS: Center for Assistive Information and Communication Solutions
ESEP: Escola Superior de Enfermagem do Porto
FES-I: Falls Efficacy Scale–International
IADL: Lawton Instrumental Activities of Daily Living Scale
IMU: inertial measurement unit
MMSE: Mini Mental State Examination
OEP: Otago Exercise Program
STEADI: Stopping Elderly Accidents, Deaths, and Injuries
SUS: System Usability Scale
TUG: Timed Up and Go
